# Gait symmetry and hip strength in women with developmental dysplasia following hip arthroplasty compared to healthy subjects: A cross-sectional study

**DOI:** 10.1371/journal.pone.0193487

**Published:** 2018-02-23

**Authors:** Ruud A. Leijendekkers, Marco A. Marra, Sjoerd Kolk, Geert van Bon, B. Wim Schreurs, Vivian Weerdesteyn, Nico Verdonschot

**Affiliations:** 1 Radboud university medical center, Department of Orthopedics, Physical Therapy, Nijmegen, the Netherlands; 2 Radboud university medical center, Orthopedic Research Laboratory, Radboud Institute for Health Sciences Nijmegen, Nijmegen, the Netherlands; 3 Radboud university medical center, Department of Rehabilitation, Donders Institute for Neuroscience, Nijmegen, the Netherlands; 4 Radboud university medical center, Department of Orthopedics, Nijmegen, the Netherlands; 5 Sint Maartenskliniek Research, Nijmegen, the Netherlands; 6 University of Twente, Laboratory for Biomechanical Engineering, Enschede, the Netherlands; University of Zaragoza, SPAIN

## Abstract

**Introduction:**

Untreated unilateral developmental dysplasia of the hip (DDH) results in asymmetry of gait and hip strength and may lead to early osteoarthritis, which is commonly treated with a total hip arthroplasty (THA). There is limited knowledge about the obtained symmetry of gait and hip strength after the THA. The objectives of this cross-sectional study were to: a) identify asymmetries between the operated and non-operated side in kinematics, kinetics and hip strength, b) analyze if increased walking speed changed the level of asymmetry in patients c) compare these results with those of healthy subjects.

**Methods:**

Women (18–70 year) with unilateral DDH who had undergone unilateral THA were eligible for inclusion. Vicon gait analysis system was used to collect frontal and sagittal plane kinematic and kinetic parameters of the hip joint, pelvis and trunk during walking at comfortable walking speed and increased walking speed. Furthermore, hip abductor and extensor muscle strength was measured.

**Results:**

Six patients and eight healthy subjects were included. In the patients, modest asymmetries in lower limb kinematics and kinetics were present during gait, but trunk lateral flexion asymmetry was evident. Patients’ trunk lateral flexion also differed compared to healthy subjects. Walking speed did not significantly influence the level of asymmetry. The hip abduction strength asymmetry of 23% was not statistically significant, but the muscle strength of both sides were significantly weaker than those of healthy subjects.

**Conclusions:**

In patients with a DDH treated with an IBG THA modest asymmetries in gait kinematics and kinetics were present, with the exception of a substantial asymmetry of the trunk lateral flexion. Increased walking speed did not result in increased asymmetries in gait kinematics and kinetics. Hip muscle strength was symmetrical in patients, but significantly weaker than in healthy subjects. Trunk kinematics should be included as an outcome measure to assess the biomechanical benefits of the THA surgery after DDH.

## Introduction

The anatomic definition of hip dysplasia refers to inadequate development of the femoral head, the acetabulum, or both [1]. If diagnosed early and treated according to a fixed protocol, the prognosis of developmental dysplasia of the hip (DDH) is good, but if diagnosed late or left untreated (neglected) it will often lead to early secondary osteoarthritis, especially in (sub-)luxated DDH [1–3]. One of the treatment options in adults is total hip arthroplasty (THA) combined with an anatomical reconstruction of the acetabulum [4]. To prevent nerve palsy after THA in patients with a high dislocation of the hip it is sometimes necessary to perform a subtrochanteric shortening osteotomy [5].

A recent systematic review revealed that during gait, kinematics and kinetics of THA patients with primary osteoarthritis patients in the sagittal plane differed from healthy subjects [6]. Furthermore, these authors concluded that studies reporting kinematics and kinetics in the frontal plane were rare. This is remarkable since some of the most clinically relevant and most frequently observed gait deviations in patients before and after THA (Trendelenburg and Duchenne gait) take place in the frontal plane. Studies focusing on differences between kinematics of the operated and non-operated side are rare as well [6]. These studies are important because they can identify compensatory inter-limb strategies which are potentially harmful, as an asymmetric gait can cause secondary complaints such as osteoarthritis of the contralateral limb [6,7].

Only few studies have yet reported kinematic and/or kinetic parameters in patients with osteoarthritis secondary to DDH who are treated with a THA [5,8,9]. These studies focussed on the hip joint and the pelvis during self-selected walking speed. Lai et al. concluded that kinematic and kinetic parameters of hip and pelvis in the frontal plane of the treated patients were closer to those of healthy women and differed significantly from the untreated patients. The parameters in the sagittal plane were not significantly different between treated and untreated patients, but both differed significantly from healthy women [8]. Marangoz et al. revealed that the patients had improved kinematics of the hip in the frontal plane after surgery and that they approached normal values [5]. Nie et al. found that patients had a significant lower hip joint range of motion on the operated side in sagittal and frontal plane compared to healthy subjects. The frontal plane hip range of motion of the unaffected side was significantly larger and lower compared to the operated side and compared to the healthy subjects, respectively [9]. These previous studies, however, did not report trunk kinematics and only measured at self-selected walking speed.

More demanding activities (such as walking at a higher speed) require more coordination and muscle strength [10,11] and may thus magnify potential residual asymmetries. Muscle weakness in patients with neglected DDH is associated with typical gait compensation strategies such as an unstable trunk, increased pelvic drop, increased lateral displacement of the pelvis, decreased hip extension, and decreased internal hip abductor moment [12,13]. Limited research has been done concerning muscle strength in patients with DDH after THA [14,15]. Yilmaz et al. assessed hip flexor and extensor muscle strength in young patients with DDH after THA and found asymmetry in flexor strength [14]. Liu et al. found asymmetry in hip abductor strength as a result of a weaker affected side in patients with DDH, both before and after THA surgery [15]. The level of asymmetry was lower after surgery. To our knowledge, there are no published studies which assessed hip muscle strength in patients with DDH in comparison to healthy subjects.

In this investigation we present the postoperative gait parameters and hip muscle strength of women with unilateral DDH after a cemented THA with impaction bone grafting of the cup (IBG) [16]. Specifically, gait parameters of hip, pelvis and trunk in the frontal and sagittal plane were assessed during walking at self-selected comfortable walking speed (CWS) and increased walking speed (CWS+30%). Furthermore, hip abduction and extension strength were evaluated. We compared the results between the operated and non-operated side and between patients and healthy subjects. We hypothesized that patients had an asymmetrical gait and that the level of asymmetry increased at increased walking speed. Additionally, we hypothesized that patients had an asymmetrical hip muscle strength with weaker muscles at the operated side. Furthermore, we hypothesized that parameters of the non-operated side better match the reference values of the healthy subjects than the parameters of the operated side.

## Materials and methods

The reporting of this cross-sectional study was done following the STROBE statement [17].

### Participants

Patients were recruited in one university hospital in the Netherlands (Radboud University Medical Center). Women between 18–70 year with unilateral DDH who had undergone a unilateral THA and had a body mass index between 17 and 30 were eligible for inclusion. Patients were excluded if they had substantial neurologic or musculoskeletal disorders that would adversely affect their ability to perform activities of daily living. The sample of age and sex-matched healthy subjects was recruited from a database of volunteers available at the department.

The study procedure was approved by the local medical ethical committee of the region Arnhem-Nijmegen, the Netherlands (study code 2012/170). Before the assessment written informed consent was obtained from each participant.

### Surgical technique

**Fig 1:**
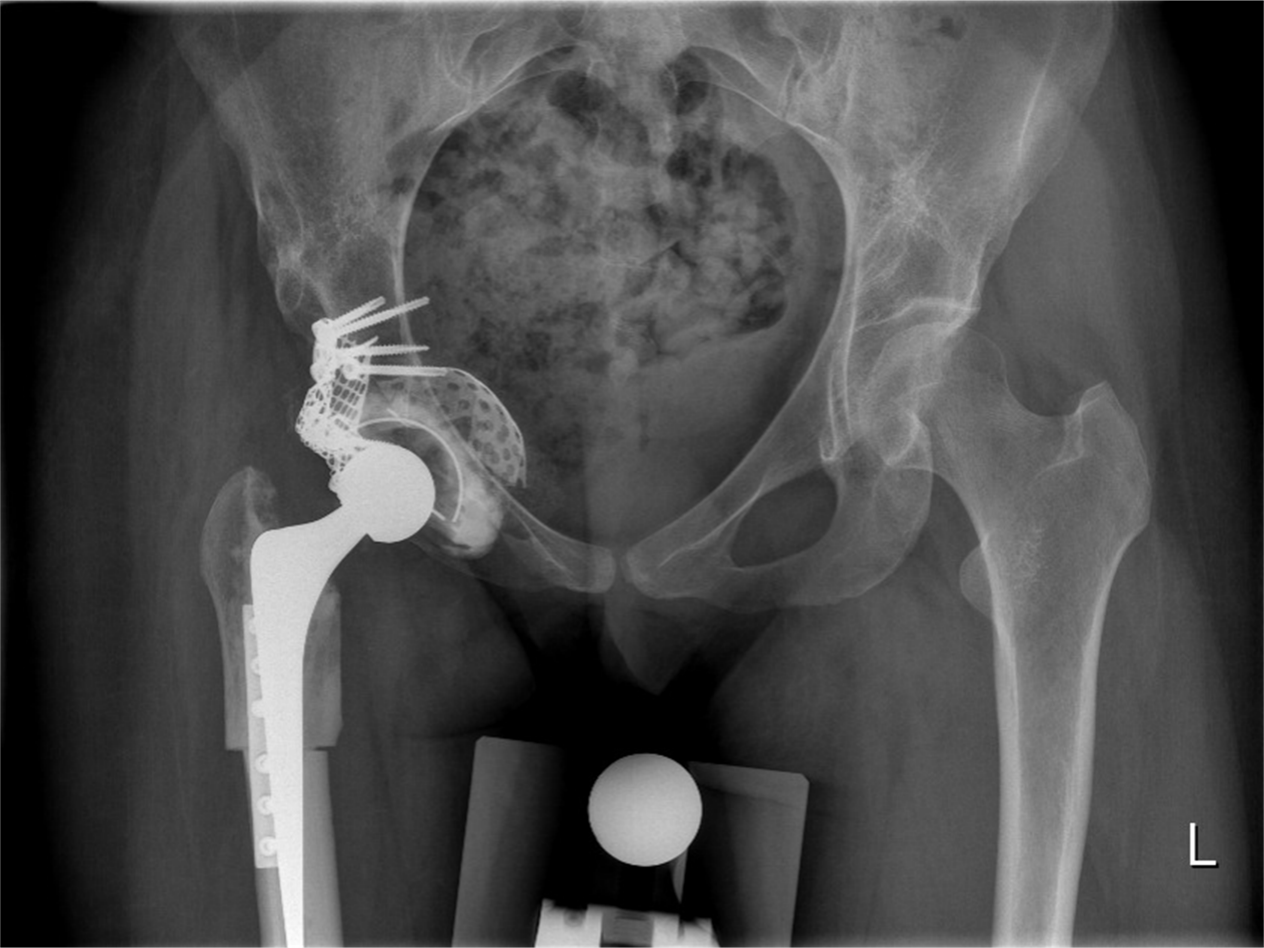
Total hip arthroplasty with impaction bone grafting of the cup (IBG) and subtrochanteric shortening osteotomy.

The THA approach was posterolateral with an anatomical reconstruction of the acetabulum using impaction bone grafting (IBG) [16]. In this procedure, medial and peripheral wall defects were closed by a metal mesh after reaming the acetabulum. The anatomical center of rotation was reconstructed with morsellized bone grafts, made from a femoral head auto- or allograft, which were compressed by impactors and a hammer. Thereafter a polyethylene contemporary cup by Stryker (Kalamazoo, United States) was inserted with bone cement. In cases of high dislocation of the hip a subtrochanteric shortening osteotomy [5]. (Fig 1) was performed before inserting the cemented Exeter stem (Stryker).

### Setup and equipment

**Fig 2:**
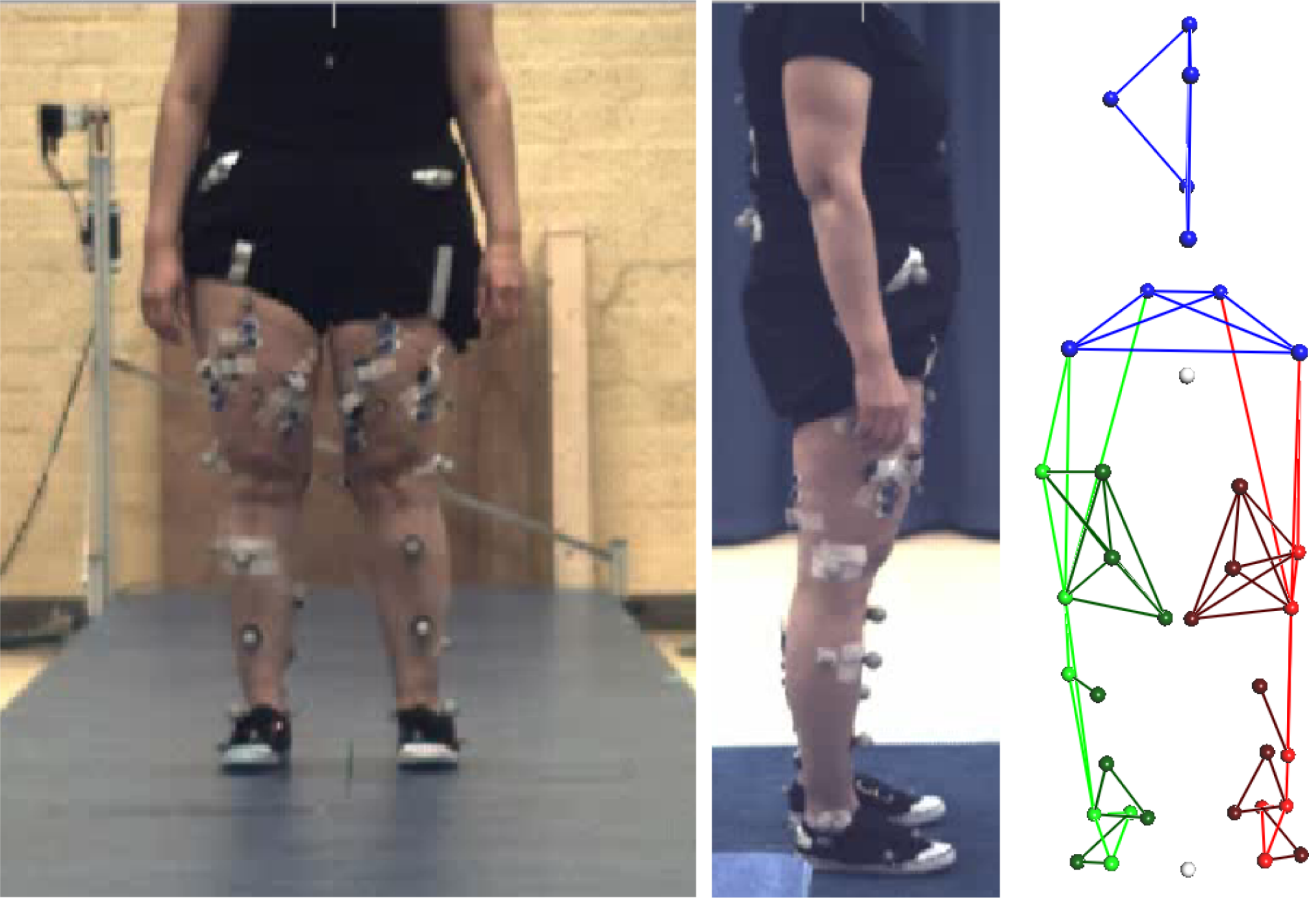
Experimental setup for gait measurements. Left: position of the Vicon markers in frontal plane, Middle: position of the Vicon markers in sagittal plane; Right: skeletal structure generated by the Vicon system (frontal plane view).

Kinematic and kinetic data were captured with a three-dimensional motion capture system (Vicon MX, Oxford, United Kingdom) containing six 100 Hz digital optical cameras and two synchronized custom made force plates (AMTI, Watertown, MA, USA) embedded in the laboratory floor which measured ground reaction forces at 1000 Hz. Thirty-five retro-reflective markers were attached to each subject following the standard Vicon Plug-in-Gait marker set, excluding the head and arm markers and expanded with additional markers placed on the anterior side of the thigh and lower leg at 1/3 and 2/3 of the segment length, and on the fifth metatarsal head of the foot (Fig 2) [18]. For the data analysis of the gait measurements we used Vicon Nexus software (version 1.8.4, Vicon MX, Oxford, United Kingdom), AnyBody Modeling System (AMS, version 6.0.5, AnyBody Technology A/S, Aalborg, Denmark) [19] and MATLAB (Release 2015b, The MathWorks Inc., Natick, Massachusetts, United States). Hip abduction and extension strength was measured with a force transducer, MATLAB was used for the data analysis.

### Study procedures and parameters

The participants were invited to the gait laboratory of our medical center for a series of assessments. First, demographic data and participant characteristics were collected. In addition, patient’s hip function and experienced hip problems were examined using the Harris Hip Score (range 0–100) [20] and the Oxford Hip Score (range 0–48), respectively [21,22]. Then, hip muscle strength was measured followed by gait analysis as described below. All measurements were performed by the same experienced researcher (SK) and participants were offered sufficient time to rest before beginning the various tests to prevent measurement bias. Each patient’s pre-operative stage of DDH (according to the system of Eftekhar [23]) was extracted from the medical files and radiographs.

#### Gait measurement

Participants walked with their own shoes, which in case of leg-length discrepancy corrected for the discrepancy, on an 8-meter long walkway at self-selected comfortable walking speed (CWS) and externally-paced at a walking speed 30% faster than CWS (CWS+30%). A winch cable with a reference point was used to pace CWS+30%. To prevent participants from targeting the force plates, they were instructed to ‘walk naturally’ and not look down at the force plates. A trial was considered correct if each foot cleanly struck one of the two force plates. The assessment ended when six correct trials were captured.

#### Data analysis gait measurement

Vicon Nexus software was used to process the gait data. Gait events (heel-strikes and toe-offs) were automatically identified using the built-in function ‘Detect gait cycle events’, based on force plate data.

**Fig 3:**
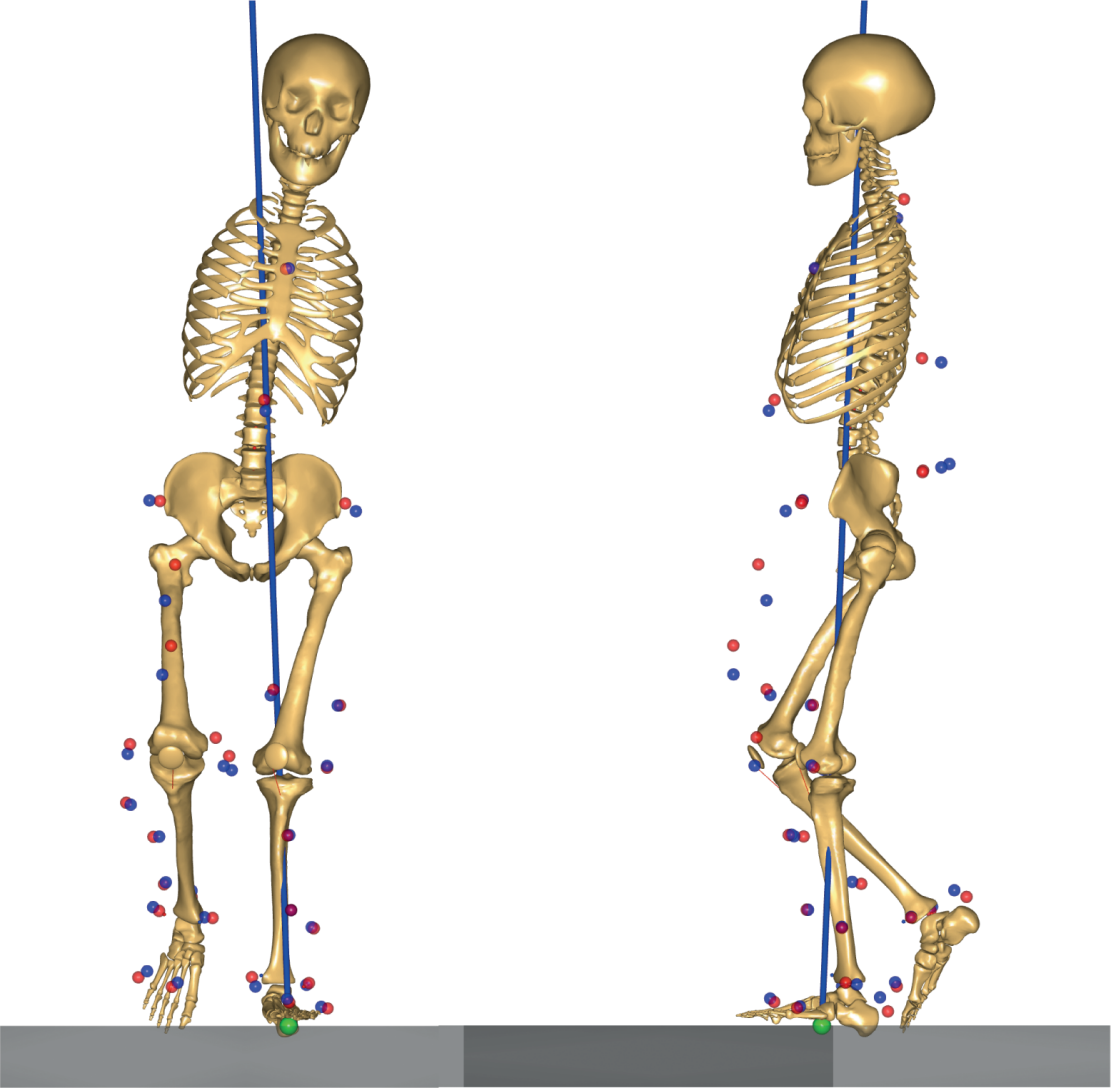
Musculoskeletal model generated by the AnyBody Modeling System. Example of a participant with a THA on the left side; Blue markers: the experimental markers recorded with the Vicon system; Red markers: the AnyBody model markers, which track the experimental markers to simulate the desired motion.

The AnyBody Modeling System [19] was utilized to calculate the kinematic and kinetic parameters of hip, pelvis and trunk in the frontal and sagittal planes during walking at CWS and CWS+30%. A 21-degree-of-freedom rigid-body musculoskeletal model was employed (Fig 3), based on the `GaitLowerExtremityModel' available in the AnyBody Managed Model Repository (version 1.5.1). The lower limb model was based on the Twente Lower Extremity Model dataset [24]. The model consisted of trunk, pelvis, thigh, shank, talus and foot segments. The body height and mass were used to linearly scale the anthropometric and inertial parameters of the model to each subject. Subsequently, marker trajectories from a reference static calibration trial, recorded for each subject, were used to further optimize the pelvis width, and the thigh, shank, and foot lengths using a parameter identification algorithm [25]. All successive analyses performed on the same subject used the pre-calculated model parameters. The variables of interest were joint kinematics and internal joint moments. For this, detailed modelling of the individual muscle-tendon units was not necessary; therefore, the model joints were equipped with ideal torque generators. Both marker and force-plate data were first filtered using a 2^nd^ order zero-phase Butterworth low-pass filter with a cut-off frequency of 12 Hz. A motion optimization algorithm [26] was used to calculate full-body kinematics, specifically: pelvis-trunk-head angles, pelvis position and orientation, hip flexion, hip abduction and hip external rotation, knee flexion, ankle plantar flexion and subtalar eversion. Subsequently, the obtained full-body kinematics were input, together with the force-plate data, to an inverse-dynamic analysis for the calculation of the internal joint moments. Pelvis and trunk angles were calculated relative to the global reference frame, whereas hip joint angles and moments were expressed according to the International Society of Biomechanics (ISB) recommendations on the joint local reference frames [27].

Peak values of frontal and sagittal plane kinematics and kinetics were extracted using MATLAB. Frontal plane kinematics and kinetics were analyzed during the single limb support phase (mid-stance: 12% to 31% of the gait cycle and terminal stance: 31% to 50% of the gait cycle) [28]. For the hip joint, adduction, flexion and extension angles, abductor moment both during mid-stance and terminal stance, flexion and extension moments were assessed. The frontal plane kinematics of the pelvis present an M-shaped curve during single limb support phase [29,30] beginning with a pelvic contralateral drop (mid-stance) followed by a (relative) pelvic hike (terminal stance) and ending with a pelvic contralateral drop (terminal stance). The (relative) pelvic hike, elevation of the contralateral side of the pelvis resulting in a pelvic obliquity closer to the neutral horizontal plane, is a strategy to facilitate foot clearance of the swing limb [28]. We assessed the pelvic contralateral drop angle during the mid-stance, the (relative) pelvic hike angle and the pelvic anterior tilt angle. For the trunk, we assessed the obliquity angle in the frontal plane, during both mid-stance and terminal stance, and the trunk flexion angle.

The joint internal moments were normalized by the body weight (BW) and height (Ht) of the subject and expressed as a percentage of their product (%BW*Ht). For each participant, the walking speed, kinematic and kinetic parameters obtained from all available trials were averaged. The subject averages were used to calculate group averages for both the patients and healthy subjects groups.

#### Muscle strength measurement

**Fig 4:**
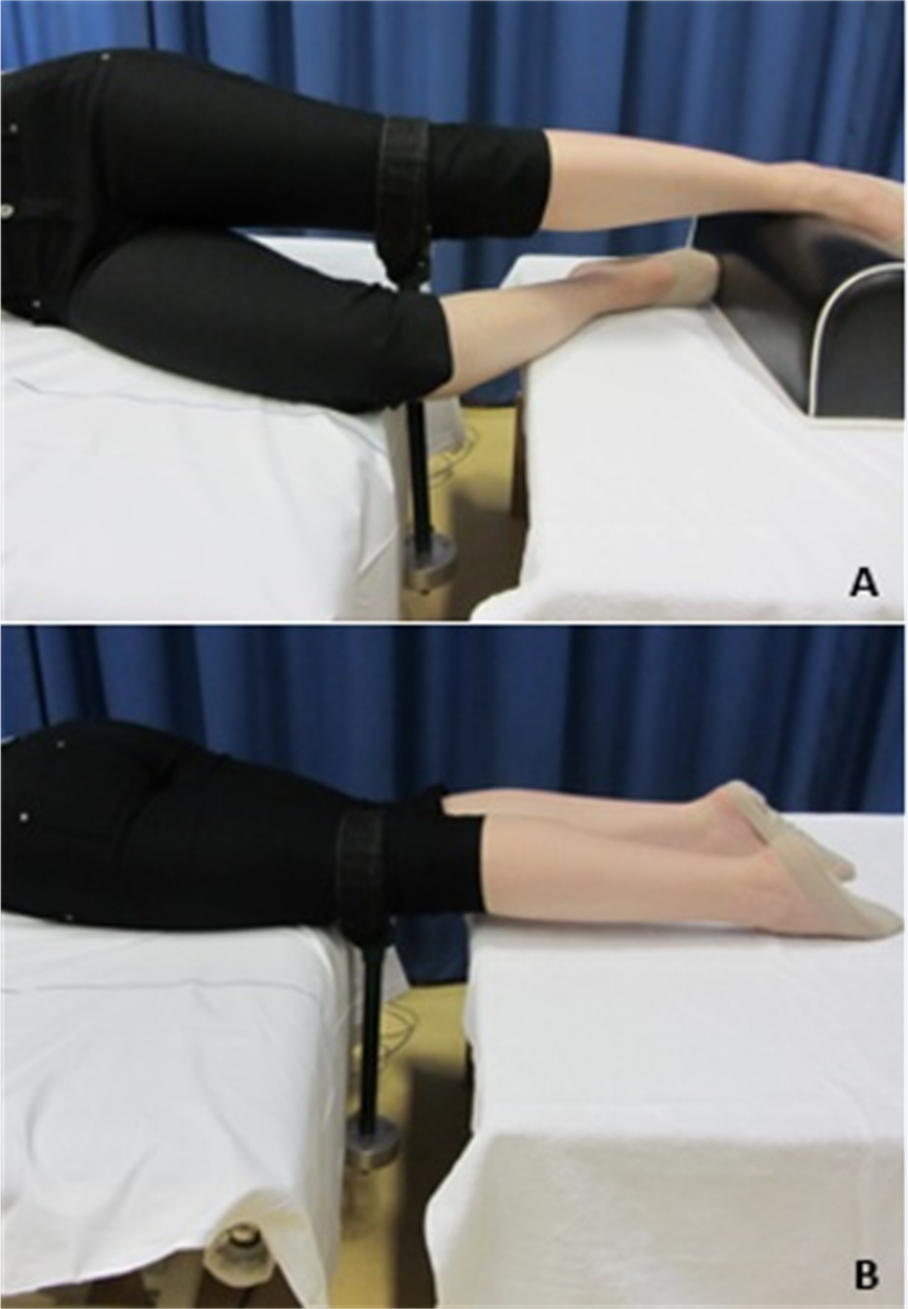
Experimental setup for muscle strength measurements. A: Hip abduction strength setup, in side-lying position. B: Hip extension strength setup, in prone position.

Isometric maximum voluntary contractions were recorded for hip abduction and extension using a force transducer (Fig 4). Hip abduction strength was tested in side-lying position, with the tested hip at 0° flexion and 0° adduction, and the knee extended. The non-tested hip was flexed at 45°, and the knee was flexed at 90° in order to prevent the contralateral limb from contributing to the maximum strength effort [18]. Hip extension was tested in supine position, with legs in neutral position.

A soft, yet non-elastic, Velcro strap was attached just proximal to the femoral epicondyles perpendicular to the limb. The transducer was rigidly attached to the testing bench (Fig 4). Participants received a standardized verbal encouragement while performing three maximum voluntary contractions for 4 seconds. Between the tests, participants rested 30 seconds to recover. Raw data was low-pass filtered at 6 Hz with a 2nd order Butterworth filter in MATLAB. The final outcome of the test was the highest recorded maximum force in Newton. Peak torque values were calculated by multiplying the maximum force by the moment arm measured from the joint axis of rotation to the point of the force application. The peak torque was normalized by body weight.

### Statistical analysis

Analyses were done in SPSS 20.0 (SPSS Inc., Chicago, Illinois, United States). All statistical tests were 2-sided, and differences with a p-value < 0.05 were considered to be statistically significant. All data were checked for normality. We used Wilcoxon signed-rank test to evaluate within-group differences. Between group differences were evaluated with the Mann-Whitney U test. Demographics, participant characteristics and walking speed were statistically tested to reveal group differences. Within the analyses of the gait parameters (kinematics and kinetics) and muscle strength the mean of the left and right side of the healthy subjects was compared to both the operated and non-operated side of the patients.

#### Gait parameters

For patients, the extent of asymmetry in kinematic and kinetic parameters was calculated as the difference between the parameter value obtained from the operated side and the corresponding value from the non-operated side, resulting in a symmetry value [31]. A symmetry value of zero means perfect symmetry. The one-sample Kolmogorov-Smirnov test was used to test whether the symmetry values differed statistically from a distribution with a mean of 0 and standard deviation of 1. Furthermore, the difference between the CWS symmetry value and the CWS+30 symmetry value was statistically tested. Comparison of the kinematic and kinetic parameters between patients and healthy subjects was done using descriptive statistics by reporting the difference in mean values. In the text we report those parameters where ≥ 80% of the patients fell outside the range of the healthy.

#### Muscle strength

For patients, the extent of asymmetry in hip abduction and hip extension strength was calculated as the difference between the parameter value obtained from the operated side and the corresponding value from the non-operated side. For patients the difference in muscle strength between operated and non-operated side was statistically tested. Further, the differences in muscle strength of both the operated and non-operated side of the patients with the healthy subjects was statistically tested.

## Results

### Demographics, participant characteristics and walking speed

Forty-seven patients and eight healthy subjects were screened for eligibility of which thirteen patients and eight healthy subjects were approached to participate in this study. Hereof, six patients and eight healthy subjects were included. In the patients, the pre-operative DDH stage was class A in three cases, class B in two cases and class C in one case according to the system of Eftekhar [23]. No significant group differences (Table 1) were found for age, weight, height, BMI or walking speed. The participating patients were between 12 and 115 months post-surgery. In one patients, the THA was combined with a subtrochanteric shortening osteotomy. The average Harris hip scores and Oxford hip scores in the patient group were ‘good’ with 85 points (range 69–94) and 40 points (range 32–46), respectively (Table 1).

**Table 1:** Demographics, participant characteristics and walking speed.

	Patients	Healthy	P-value
	(n = 6)	subject	
		(n = 8)	
Stage of developmental dysplasia of the	A: 3	NA	NA
hip before surgery (according to the	B: 2		
system of Eftekhar)	C: 1		
Age at assessment (years)	40.7 (13.0)	33.0 (10.4)	0.181
Weight (kg)	63.8 (5.5)	66.2 (11.6)	1.000
Height (m)	1.64 (0.07)	1.72 (0.07)	0.181
BMI (kg/m^2^)	23.9 (3.1)	22.3 (3.0)	0.345
Time since surgery (months)	49.8 (54.1)	NA	NA
Operated side	L: 4, R: 2	NA	NA
Subtrochanteric shortening osteotomy	Yes: 1, No: 5	NA	NA
Harris Hip Score (0–100 points)	85 (10)	NA	NA
Oxford Hip Score (0–48 points)	40 (6)	NA	NA
CWS (m/sec)	1.20 (0.22)†	1.32 (0.22)	0.435
CWS+30% (m/sec)	1.56 (0.29)†	1.61 (0.26)	1.000

BMI: Body Mass Index; CWS: self-selected comfortable walking speed; CWS+30%: walking 30% faster than CWS; L: left; R: right; NA: Not Applicable; Continuous data is presented as mean (SD), categorical data as numbers; Statistical tests used: Mann-Whitney U test

*: Values considered significant.

†: Based on n = 5.

### Gait

The kinematic and kinetic parameters during CWS and CWS+30 of both the patients and the healthy subjects are presented in Table 2. One patient was excluded from the calculation and analyses of the kinematic and kinetic outcomes because the CWS+30 was only 3% faster than the CWS and CWS was an outlier (1.73 m/sec) compared to the other patients.

**Table 2:** Outcome kinematic and kinetic parameters during stance phase.

		CWS			CWS+30%		
		Patients		Healthy subjects	Patients		Healthy subjects
		Operated side mean (SD)	Non-operated side mean (SD)	mean (SD)	Operated side mean (SD)	Non-operated side mean (SD)	mean (SD)
**Kinematic parameters**							
Peak hip adduction							
	Angle (°)	7.0 (4.0)	5.9 (3.1)^3^	7.3 (1.8)	7.1 (4.4)	5.8 (3.6)^3^	6.5 (1.8)
Peak hip flexion							
	Angle (°)	31.6 (7.9)	34.9 (6.8)	32.4 (6.4)	35.1 (9.2)	38.1 (6.9)	36.1 (6.8)
Peak hip extension							
	Angle (°)	4.4 (10.6)	5.2 (9.7)	8.7 (7.8)	7.3 (10.7)	8.2 (9.4)	11.1 (7.6)
Pelvic contralateral drop MSt							
	Angle (°)	4.3 (2.8)	2.3 (2.7)^3^	4.8 (1.5)	5.2 (3.0)	2.7 (2.4)^3^	5.0 (1.5)
Relative pelvic hike TSt							
	Angle (°)	2.2 (2.9)^3^	0.3 (2.6)^2^	3.0 (1.2)	2.6 (3.3)^3^	0.4 (2.7)^2^	3.1 (1.0)
Peak pelvic anterior tilt							
	Angle (°)	12.2 (6.8)^3^	12.4 (7.0)^3^	6.6 (4.5)	13.8 (6.4)	13.6 (6.8)	8.1 (4.6)
Peak trunk obliquity frontal plane							
	Angle MSt (°)	7.5 (2.6)^1^	-0.7 (1.0)^1^	1.7 (0.9)	7.7 (2.5)^2^	-0.5 (1.2)^1^	2.5 (1.3)
	Angle TSt (°)	7.7 (2.4)^1^	0.5 (1.3)^2^	2.9 (0.4)	7.6 (2.6)^1^	0.2 (1.1)^1^	3.1 (0.6)
Peak trunk flexion							
	Angle (°)	-0.7 (4.5)	-0.8 (4.4)	0.8 (3.3)	-0.5 (3.5)	-0.5 (3.6)	1.5 (4.0)
**Kinetic parameters**							
Peak hip abductor moment							
	MSt (%BW*Ht)	3.4 (0.6)^3^	3.7 (0.9)	4.0 (0.5)	3.4 (0.7)^3^	3.9 (1.2)	4.1 (0.5)
	TSt (%BW*Ht)	3.1 (0.8)^3^	3.2 (1.1)	4.0 (0.8)	2.9 (0.7)^3^	3.1 (1.5)	3.6 (0.8)
Peak hip flexion moment							
	(%BW*Ht)	2.6 (1.0)	2.4 (0.7)	3.1 (1.4)	3.8 (1.5)	3.8 (1.3)	4.4 (1.7)
Peak hip extension moment							
	(%BW*Ht)	4.6 (1.3)	5.2 (0.8)	4.8 (1.6)	6.2 (1.7)	6.6 (0.8)	6.2 (2.3)

CWS: self-selected comfortable walking speed; CWS+30%: walking 30% faster than CWS; Symmetry value: difference between the two sides (operated side minus non-operated side); MSt: Mid-Stance; TSt: Terminal Stance; Trunk obliquity frontal plane: positive values represent ipsilateral lateral flexion, negative values represent contralateral lateral flexion; Trunk flexion: positive values represent trunk flexion, negative values represent trunk extension; Joint moments given as internal moments in %BW*Ht: moments normalized by the body weight (BW) and height (Ht) and expressed as a percentage of their product (%BW*Ht); Outcomes of 100% (^1^), 80% (^2^) or 60% (^3^) of the patients were outside the outcome range of the healthy subjects.

#### Kinematic parameters

Most symmetry values in sagittal plane (Table 3) were below one degree with the exception of hip flexion which presented asymmetry as a result of smaller hip flexion (CWS: 3.3° (2.0) and CWS+30: 3.1° (2.7)) on the operated side. Both symmetry values differed statistically from zero (Table 3). In the frontal plane (Table 3) all parameters had symmetry values higher than one degree. The frontal plane values of the operated side were greater than of the non-operated side (Table 2). Only the symmetry values of the ipsilateral trunk lateral flexion (CWS: 7.2° (3.2) to 8.2° (2.7) and CWS+30: 7.4° (3.2) to 8.2° (2.9)) differed statistically from zero (Table 3). No significant differences in symmetry values between CWS and CWS+30 were found (Table 3).

**Table 3:** Kinematic and kinetic symmetry values of the patients.

		CWS	CWS+30	CWS versus CWS+30	
		Symmetry value mean (SD)	Symmetry value mean (SD)	Differences in symmetry value mean (SD)	95% Confidence Interval
**Kinematic parameters**					
Peak hip adduction					
	Angle (°)	1.2 (5.9)	1.2 (6.5)	-0.1 (1.4)	-1.8; 1.7
Peak hip flexion					
	Angle (°)	-3.3 (2.0)*	3.1 (2.7)*	-0.2 (1.2)	-1.0; 0.7
Peak hip extension					
	Angle (°)	-0.8 (6.6)	-0.9 (7.1)	-0.2 (0.7)	-1.7; 1.2
Pelvic contralateral drop MSt					
	Angle (°)	1.9 (5.0)	2.5 (5.0)	-0.6 (0.9)	-1.7; 0.5
Relative pelvic hike TSt					
	Angle (°)	1.9 (5.4)	2.2 (5.8)	-0.3 (1.6)	-2.3; 1.7
Peak pelvic anterior tilt					
	Angle (°)	-0.2 (0.7)	0.2 (1.2)	-0.3 (0.7)	-1.2; 0.6
Peak trunk obliquity frontal plane					
	Angle MSt (°)	8.2 (2.7)*	8.2 (2.9)*	-0.1 (0.8)	-1.1; 0.9
	Angle TSt (°)	7.2 (3.2)*	7.4 (3.2)*	-0.2 (0.7)	-1.0; 0.6
Peak trunk flexion					
	Angle (°)	0.2 (0.4)	0.0 (0.2)	0.1 (0.3)	-0.2; 0.5
**Kinetic parameters**					
Peak hip abductor moment					
	MSt (%BW*Ht)	-0.3 (0.8)	-0.5 (1.1)	0.2 (0.5)	-0.4; 0.7
	TSt (%BW*Ht)	-0.1 (0.7)	-0.2 (1.0)	0.1 (0.7)	-0.7; 1.0
Peak hip flexion moment					
	(%BW*Ht)	0.2 (0.5)	0.0 (0.6)	0.3 (0.3)	-0.1; 0.6
Peak hip extension moment					
	(%BW*Ht)	-0.5 (0.9)	-0.4 (1.1)	0.1 (0.4)	-0.3; 0.6

CWS: self-selected comfortable walking speed; CWS+30%: walking 30% faster than CWS; Symmetry value: difference between the two sides (operated side minus non-operated side); MSt: Mid-Stance; TSt: Terminal Stance; Joint moments given as internal moments in %BW*Ht: moments normalized by the body weight (BW) and height (Ht) and expressed as a percentage of their product (%BW*Ht); Statistical tests used: one-sample Kolmogorov-Smirnov test to test whether the symmetry values differed statistically from 0 (* in the first two columns represent a statistical difference), Wilcoxon signed-rank test for the comparison of CWS and CWS+30.

**Fig 5:**
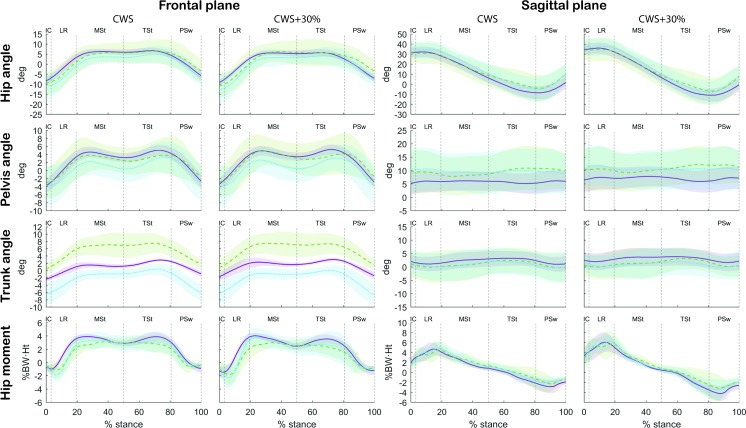
Kinematics in frontal and sagittal plane of women with unilateral DDH after a cemented THA and healthy subjects. Green/dashed: operated side of patients; blue/dotted: non-operated side of patients; Purple/solid line: healthy subjects. The shaded areas represent the 95% confidence intervals of the mean. The kinematic and kinetic data were normalized to the stance phase of the gait cycle (heel strike to ipsilateral toe off). IC: Initial Contact; LR: Loading Response; MSt: Mid-Stance; TSt: Terminal Stance; PSw: Pre-Swing; deg: degrees; BW: body weight; Ht: height. Hip angle frontal plane: positive values represent adduction, negative values represent abduction; Hip angle sagittal plane: positive values represent flexion, negative values represent extension; Pelvis angle frontal plane: positive values represent pelvic contralateral drop, negative values represent pelvic hike; Pelvis angle sagittal plane: positive values represent pelvic anterior tilt, negative values represent pelvic posterior tilt; Trunk angle frontal plane: positive values represent ipsilateral lateral flexion, negative values represent contralateral lateral flexion; Trunk angle sagittal plane: positive values represent trunk flexion, negative values represent trunk extension; Hip moment frontal plane: positive values represent an abductor internal moment, negative values represent an adductor internal moment; Hip moment sagittal plane: positive values represent an extension moment, negative values represent a flexion moment.

Comparison of the patients with the healthy subjects (Table 2 and Fig 5) showed that the most evident differences were present in the frontal plane. The non-operated side of 80% of the patients presented a greater relative pelvic hike during terminal stance (difference CWS: 2.7° and CWS+30: 2.7°) than the healthy subjects. Trunk lateral flexion during both the stance phase of the operated and non-operated side clearly differed from the healthy subjects in 80–100% of the patients. The peak trunk lateral flexion angle during the stance phase of the non-operated side seemed to better resemble those of the healthy subjects, because the difference was smaller compared to the operated side (difference with operated side CWS: 4.8° to 9.2° and CWS+30: 4.5° to 5.2°; non-operated side CWS: -2.4° and CWS+30: -2.9° to -3.0°), however the direction of the movement was reversed during mid-stance. Healthy subjects presented a small ipsilateral lateral flexion (CWS: 1.7° to 2.9° and CWS+30: 2.5° to 3.1°). The non-operated side presented a smaller ipsilateral lateral flexion during terminal stance (CWS: 0.5° and CWS+30: 0.2°) and even a contralateral trunk lateral flexion during mid-stance (CWS: 0.7° and CWS+30: 0.5°) while the operated side presented a greater ipsilateral lateral flexion during both the mid-stance and the terminal stance (CWS: 7.5° to 7.7° and CWS+30: 7.6° to 7.7°) than the healthy subjects.

#### Kinetic parameters

The mid-stance hip abductor and peak hip extension moment on the operated side were generally ~10% smaller than on the non-operated side, yet these differences did not reach significance (CWS: -0.3 (0.8) %BW*Ht to -0.5 (0.9) %BW*Ht and CWS+30: -0.4 (1.1) %BW*Ht to -0.5 (1.1) %BW*Ht; Table 3).

None of the symmetry values differed statistically from zero (Table 3). No statistically significant differences in symmetry values between CWS and CWS+30 were found (Table 3).

Comparison of the patients with the healthy subjects showed a very similar pattern in the sagittal plane and a slightly different pattern in the frontal plane (Table 2 and Fig 5). None of the parameters had outcomes in which equal or more than 80% of the patients were outside the outcome range of the healthy subjects.

### Muscle strength

Due to technical problems during data collection, muscle strength outcomes of one patient were missing. Hip abduction strength was on average 23% lower on de operated side compared to the non-operated side (difference of -0.3 (0.3) Nm/kg), yet this difference did not reach significance (Table 4). Hip extension strength did not show evident asymmetry between operated and non-operated side (0.1 (0.4) Nm/kg). The hip abduction and extension strength of the patient was significantly lower than of the healthy subjects. The hip abduction strength of the operated side was 0.9 Nm/kg lower (45%) and the non-operated side was 0.7 Nm/kg lower (35%). The hip extension strength of the operated side was 0.8 Nm/kg lower (40%) and the non-operated side was 0.9 Nm/kg lower (45%).

**Table 4:** Muscle strength.

		Patients mean (SD)	Healthy subjects mean (SD)	Patients	Patients versus healthy subjects	
				P-value (O versus N)	P-value (O versus healthy subjects)	P-value (N versus healthy subjects)
Hip abduction peak torque						
	O (Nm/kg)	1.1 (0.4)	2.0 (0.4)	0.138	0.003*	0.003*
	N (Nm/kg)	1.3 (0.1)				
	Diff (Nm/kg)	-0.3 (0.3)				
Hip extension peak torque						
	O (Nm/kg)	1.2 (0.4)	2.0 (0.3)	0.893	0.008*	0.005*
	N (Nm/kg)	1.1 (0.2)				
	Diff (Nm/kg)	0.1 (0.4)				

Isometric maximum voluntary contractions given as peak torque values in Nm/kg: Newton-meters, normalized to bodyweight in kilograms. O: operated side of patients; N: non-operated side of patients; Diff: difference between the two sides (operated side minus non-operated side); Statistical tests used: Wilcoxon signed-rank test for within-group comparison, Mann-Whitney U test for between-group comparison.

*: Values considered significant.

## Discussion

In women with unilateral DDH treated with an IBG THA, we observed modest asymmetries in lower limb kinematics and kinetics during both CWS and CWS+30. In contrast, evident asymmetry was present in the frontal plane trunk kinematics. The symmetry values of hip flexion and ipsilateral trunk lateral flexion were the only ones that differed significantly from zero, so we could not fully accept our a priori hypothesis. No significant change in symmetry of the kinematic and kinetic parameters was found as a result of an increased walking speed, which was in contrast with our a priori hypothesis.

Comparison with healthy subjects revealed several kinematic and kinetic alternations in patients, including a greater relative pelvic hike during terminal stance of the non-operated side, greater ipsilateral lateral flexion of the operated side and smaller ipsilateral lateral flexion of the non-operated side. Further, patients had lower kinetic values than healthy subjects (with the exception of the hip extension moment on the non-operated side) during both walking speeds. The kinetic values of most patients were within the outcome range of the healthy subjects, with exception of hip abductor moment on the operated side during both walking speeds. The parameters of the non-operated side did not match structurally better with the reference values of the healthy subjects, which is not in line with our a priori hypothesis.

No significant hip abduction and extension strength asymmetry was present in the patients, which altered our a priori hypotheses. Although, the hip abduction asymmetry of 23% was not statistically significant, it can be seen as a clinical relevant difference. Both sides were significantly weaker than the healthy subjects. This lower strength could result in strategies to decrease hip abductor moments such as greater hip adduction angles and ipsilateral trunk lateral flexion. This pattern was partially observed on the operated side, however, not on the non-operated side, despite the presence of bilateral muscle weakness.

A remarkable finding was that the contralateral pelvic drop on the operated side was rater similar to the healthy subjects (difference CWS: 0.5° and CWS+30: -0.2°). An increased contralateral pelvic drop was expected based on reported hip muscle weakness and previous research in the untreated DDH population [13,15]. The reversed pelvic kinematic values of the non-operated side relative to the healthy subjects, represented by a continuous oblique position of the pelvis (relative pelvic hike) during the stance phase of the non-operated-side, is similar to the findings of previous research [8,13]. The normalized contralateral pelvic drop on the operated side could be the result of the THA with IBG treatment due to lengthening of the lever arm of the abductor muscles, which results in recovery of abductor strength and reduction of the excessive contralateral drop [13,15,32]. The found level of asymmetry in hip abductor strength between the operated and the non-operated side is similar to the findings of Liu et al. [15]. A possible explanation for the hip muscle weakness of both sides (35% to 45%) compared to the healthy subject is that our population is relatively less active, this hypothesis is supported by the average Harris hip score of 85 points and the Oxford hip score of 40 points. The reduced hip angles of the operated side in sagittal plane compared to the healthy subjects corresponds with the findings of Marangoz et al. [5]. The reduced extension angle could be the result of an existing flexion contracture, which in turn induced the increased anterior pelvic tilt found in our study, and the reduced stride length found by Marangoz et al. and Lai et al. [5,8]. Remarkable is that Lai et al. found greater hip extension and smaller anterior pelvic tilt angles than in healthy subjects which is the opposite of our result [8]. Similar to our results they also found smaller hip flexion angles of the operated side compared to healthy subjects. Studies focussing on symmetry between the operated and non-operated side in subjects after THA surgery are rare [6]. We found a hip flexion asymmetry which is supported by the findings of Kiss et al. [33]. However, in their study patients after a direct-lateral approached THA for unilateral hip osteoarthritis presented an asymmetry of 12 degrees, which is larger than the 3 degrees of asymmetry we found. Benedetti et al. do not support these findings because they did not find any asymmetries in the hip joint kinematics during level walking in a similar population as included by Kiss et al. [34].

A limitation of this study was the small sample size, which obviously makes it difficult to generalize the results. However, large sample sizes in gait analysis studies using a three-dimensional motion capture system are challenging because the assessments take a considerable amount of time, especially when the study contains two tasks (CWS and CWS+30); this puts a large burden on the patients involved and requires limitation of the number of patients involved. Due to the small sample size we had a lack of power (i.e. risk of type II errors). It may, therefore, be that the more subtle asymmetries that can be observed from the symmetry values (e.g. in hip abductor and extension moments) would have become statistically significant in a larger group of patients. Yet, the clinical relevance of such findings is questionable. Due to the small sample size there is also a risk of multiple testing resulting in type I errors. To limit the amount of testing we used descriptive statistics for the comparison between the patients and the healthy subjects. The only significant results in our study were that the symmetry values of hip flexion and ipsilateral trunk lateral flexion differed significantly from zero. These symmetry values were the only ones that were clearly larger than zero. Therefore we believe that type I and II errors did not bias our results and conclusions. A second limitation is the absence of pre-operative gait analysis and muscle strength data, which limits us in making inferences on the exact benefits of the surgical intervention with regards to the walking pattern.

On the other hand, this study had a number of strong points. Firstly, the following methodological aspects are strong points because they decreased the chance of measurement bias; a) the use of gold standard tools for obtaining gait parameters and muscle strength, b) the use of a standardized method to increase the CWS with 30%. Secondly, the fact that we assessed trunk kinematics which has not been done before for this type of population. Our study points out that the most evident asymmetry in this population was present in the trunk. Therefore, trunk kinematics should be included in future research as an outcome measure to assess the biomechanical benefits of the THA surgery. Lastly, the value of this study resides also in its enhanced insight into gait parameters during different walking tasks. Although we did not find a change of asymmetry as a result of a more demanding activity, gait parameter asymmetries still may be a problem in daily living. Perhaps, our experimental tasks were not challenging enough to induce muscle fatigue and to provoke gait parameter asymmetries. So, future research could investigate gait parameters in relation to e.g. submaximal functional capacity tests, such as the 6-minute walking test (6MWT). A 6MWT reflects the functional exercise level for daily physical activities [35]. A second benefit of using a 6MWT in evaluation of THA surgery is that it evaluates a different aspect of the activity level of patients than a questionnaire such as the Harris hip score or Oxford hip score because these questionnaires measure various constructs including activities of daily living. In addition, the physical activity level in daily life should be assessed, e.g. with accelerometer monitoring. This is important because patient-reported measures, performance-based assessments and monitoring in daily life present different aspects of the activity level of patients and vary in responsiveness [36,37]. Knowledge concerning the different aspects of the activity level of patients may help to understand the cause of the reduced muscle strength of both sides in patients compared to the healthy subjects.

## Conclusion

This study showed modest asymmetries in gait kinematics and kinetics in patients with a DDH treated with an IBG THA, with the exception of a substantial asymmetry of the ipsilateral trunk lateral flexion. Increased walking speed did not result in increased asymmetries in gait kinematics and kinetics. Both the operated and non-operated side show often dissimilarities relative to the reference values of the healthy subject. Hip muscle strength was symmetrical in patients, but significantly weaker than in healthy subjects.

Despite symmetrical hip muscle weakness, the pelvis kinematics of the operated side in frontal plane were quite similar to healthy controls. It remains unclear if this is attributable to the reduction of hip abductor insufficiency as a result of the IBG THA treatment. Further research is necessary to explore the biomechanical benefits of the THA surgery in patients with DDH. It is recommended to include trunk kinematics as outcome and to use a more challenging daily living task to trigger potential insufficiencies such as muscle fatigue and gait parameter asymmetries.

## Supporting information

S1 File Gait data. (SAV)

S2 File Baseline and strength data. (SAV)
